# Microplastic Has No Effect on Rice Yield and Gaseous N Emission from an Infertile Soil with High Inorganic N Inputs

**DOI:** 10.3390/plants13091279

**Published:** 2024-05-06

**Authors:** Si Wu, Haiying Lu, Zhenghua Yi, Gui Chen, Haijun Sun

**Affiliations:** 1 Co-Innovation Center for Sustainable Forestry in Southern China, Nanjing Forestry University, Nanjing 210037, China; nlwus@njfu.edu.cn (S.W.); luhaiying@njfu.edu.cn (H.L.); yizhh@njfu.edu.cn (Z.Y.); 2 Institute of Biotechnology, Jiaxing Academy of Agricultural Science, Jiaxing 314016, China

**Keywords:** amino acid, ammonia volatilization, hybrid rice, microplastic, nitrous oxide

## Abstract

Microplastic might affect the crop yield, nitrogen (N) use efficiency and reactive N losses from agricultural soil systems. However, evaluation of these effects in infertile soil planted with different rice cultivars is lacking. We conducted a soil column experiment to determine the influence of a typical microplastic polyethylene (PE) input into an infertile soil with 270 kg N ha^−1^ and planted with two rice cultivars, i.e., a common rice Nangeng 5055 (NG) and a hybrid rice Jiafengyou 6 (JFY). The results showed that JFY produced a significantly (*p* < 0.05) greater grain yield than NG (61.6–66.2 vs. 48.2–52.5 g pot^−1^) but was not influenced by PE. Overall, PE hardly changed the N use efficiency of NG and JFY. Unexpectedly, PE significantly (*p* < 0.05) increased the total amino acid content of NG. Compared with JFY, NG volatilized significantly (*p* < 0.05) more ammonia (NH_3_) (0.84–0.92 vs. 0.64–0.67 g N pot^−1^) but emitted equal nitrous oxide (N_2_O). PE exerted no effect on either NH_3_ volatilization or the N_2_O emission flux pattern and cumulative losses of the rice growth cycle, whether with NG or JFY. Some properties of tested soils changed after planting with different rice cultivars and incorporating with microplastic. In conclusion, the rice production, N use efficiency, NH_3_ volatilization and N_2_O emission from the N-fertilized infertile soil were pronouncedly influenced by the rice cultivar, but not the PE. However, PE influenced the grain quality of common rice and some properties of tested soils with both rice cultivars.

## 1. Introduction

As a widely grown staple crop all over the world, rice provides a major food source for nearly 50% of the world population [1]. With the global population growing, farmers rely on nitrogen (N) fertilizer to enhance the rice production [2,3]. However, excessive N fertilizer application has resulted in a large quantity of reactive N (Nr) losses in rice production systems, particularly via NH_3_ volatilization and N_2_O emission [4,5]. De et al. (2007) estimated that the NH_3_ volatilization losses could account for 10–60% of the total N fertilizer applied during the rice growth season [6]. The N_2_O that was emitted from Chinese rice paddy fields also accounted for 7–11% of the total N_2_O emissions from Chinese stable croplands [7]. Therefore, evaluating and mitigating the nitrogenous gas pollutants (NH_3_ and N_2_O) are of great significance in rice production systems.

Understandably, rice yield increase mainly depends on the production per unit of the farmland area, which can be archived via hybrid rice plantations. The important basis for high yields in super rice is a large panicle with more grains, excellent structure of the canopy and strong root system [8,9]. Previous studies have demonstrated that the differences between common and hybrid rice in terms of their responses to N utilization are controlled by genetic factors as well as soil additives and N application methods [10,11]. It is widely accepted that super rice has a higher yield potential and increased N use efficiency (NUE) [11,12]. Another result also showed that the cumulative NH_3_ volatilization of hybrid rice was lower than that of common rice under the same N application rate [13].

Microplastic (MP), plastic that is ≤5 mm in diameter, has become one typical emerging pollutant due to the globally widespread use of plastic products [14]. Concerns related to microplastics in terrestrial ecosystems have been growing in recent years. No exception, agricultural soils are receptors of microplastics from many sources, including usages of coated fertilizers and plastic mulch, applications of compost and organic fertilizers and wastewater irrigation [15,16]. The amount of MPs caused by organic fertilizer application is estimated at 52 to 26,400 tons per year in Chinese agricultural soils [17]. What is more, the amount of agricultural mulch used reached nearly 15 million tons by 2015 in China, while no more than 60% of it was recycled. This resulted in a large amount of mulch residue and serious microplastic pollution [18]. As reported, microplastics have severe consequences on NUE, crop yield and soil properties [19]. The microplastic pollutants that enter the soil environment can migrate from the surface soil layer to deeper layers, which disturbs the soil microstructure and other properties [20]. In addition, MPs in the soil inactivate soil enzymes, causing changes in the abundance and diversity of soil microbiota and disordering plant metabolism [19,21]. These changes due to microplastic will further impact crop growth, yield and even the edible grain quality [19,21].

The concentration of NH_4_^+^-N and pH of surface soil or overlying water are the main factors influencing NH_3_ volatilization [22]. It had been found that the theoretical maximum capacity of NH_4_^+^-N sorption could increase from 7.1% to 17.0% while the microplastics and biochar are input together [23]. Microplastics undergo processes such as adsorption, desorption and agglomeration in the soil and act as transport media or carriers, which all influence the soil properties [24]. It can be hypothesized that MPs may affect NH_3_ volatilization and N_2_O emission by altering NH_4_^+^-N concentrations and disrupting ammonia-oxidizing microbial activities and enzymatic activities that are closely related to the N cycles in agricultural ecosystems. MP pollution in soil also affects soil properties, crop growth and nutrient utilization, like other soil additives, such as biochar and N fertilizer [21,25]. Consequently, there might be differences in the common and hybrid rice growth and N utilization when facing MP pollutants in paddy soil.

Rice cultivars and microplastic not only affect rice growth and yield but also impact the rice grain quality, especially amino acids, which are essential for human nutrition and health [26]. Therefore, rice growth and grain yield are affected by MPs; simultaneously, the content of grain amino acids may also be changed and finally lead to impaired rice quality. Moreover, various rice genes and cultivars dominate the content of amino acids in rice grain [27]. Overall, it is significant for this research to identify what the influences of MPs are on quality (taking amino acid content as an example) in common and hybrid rice.

This research analyzed the effects of a typical MP polyethylene (PE) on soil properties, grain yield and quality, NUE, NH_3_ volatilization and N_2_O emission in rice field soil with two rice cultivars. Our work also expected to clarify whether the aforementioned effects of microplastic were as a function of the rice cultivar.

## 2. Results

### 2.1. Rice Grain Yield and Amino Acid Content

Table 1 shows that the grain yields of hybrid rice were significantly (*p* < 0.05) higher than those of conventional rice (61.6–66.2 vs. 48.2–52.5 g pot^−1^). Regarding the yield components, the hybrid rice had significantly (*p* < 0.05) higher kernels per spike (average 107%), but with a significantly (*p* < 0.05) lower spike number and thousand grain weight (average 21% and 13%, respectively). Moreover, hybrid rice recorded higher plant height and produced more straw biomass than rice treatments (74.3–78.2 vs. 67.2–71.5 cm and 34.9–36.8 vs. 29.2–32.5 g pot^−1^, respectively). Microplastic PE did not influence the plant height, straw biomass, grain yield and its components of rice for the same cultivar. Nevertheless, microplastic PE exerted a potentially inhibiting effect on the grain yield of both rice cultivars. The grain yields were 8.2% and 6.9% lower in NG + PE and JFY + PE than NG and JFY, respectively, though the differences were not statistically significant (*p* > 0.05).

**Figure 1:**
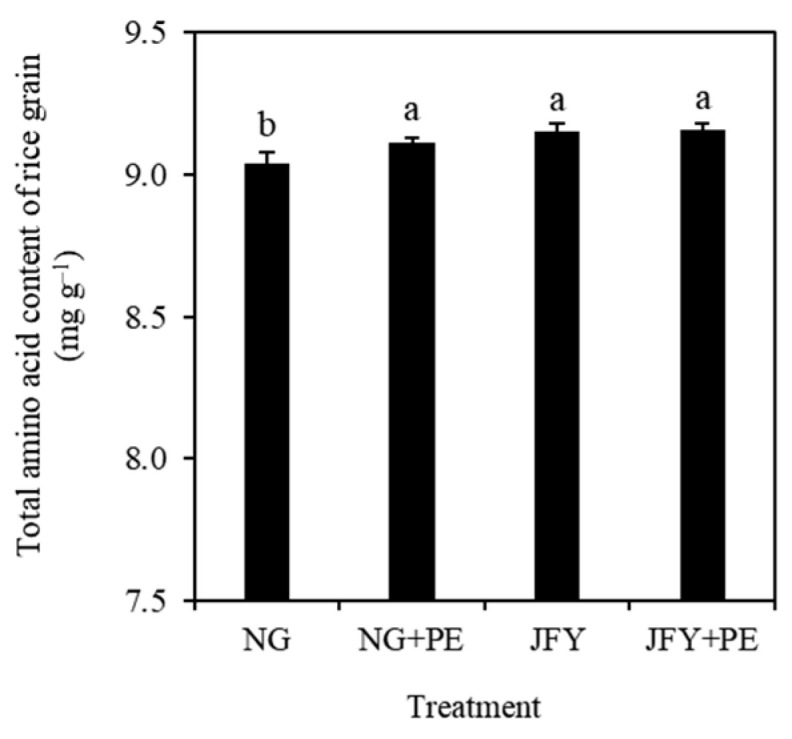
Effects of PE on the total amino acid content in the grain of a conventional rice (NG) and a hybrid rice (JFY). Different bars indicate the SD of means (*n* = 3), and lowercase letters above the column indicate that the differences among treatments are significant at *p* < 0.05.

Without PE, hybrid rice grain contained a significantly (*p* < 0.05) higher total amino acid content (1.3%) than conventional rice grain (Figure 1). For hybrid rice, PE addition exerted no influence on the total amino acid content of grain. Interestingly, for conventional rice, the total amino content of NG + PE was significantly higher (*p* < 0.05, 0.8%) than that of the NG treatment.

### 2.2. Nitrogen Use Efficiencies

As can be seen from Table 2, for both conventional and hybrid rice, whether PE existed or not, their N recovery efficiency (REN) and N agronomic efficiency (AEN) were equal. The N physiology efficiency (PEN) of conventional rice was significantly (*p* < 0.05) lower than that of hybrid rice (52.3–57.0 vs. 64.0–65.9 g grain g^−1^ N), either with or without PE addition. The PEN of conventional rice was significantly (*p* < 0.05) decreased by 8.2% with PE addition, whose effect was not found for hybrid rice. Without PE addition, the partial factor productivity of N (PFPN) of JFY rice was significantly higher (*p* < 0.05, 26.0%) than that of NG. Nevertheless, PE did not exert influence on the PFPN of both rice cultivars.

### 2.3. NH_3_ Volatilization and N_2_O Emission

**Figure 2:**
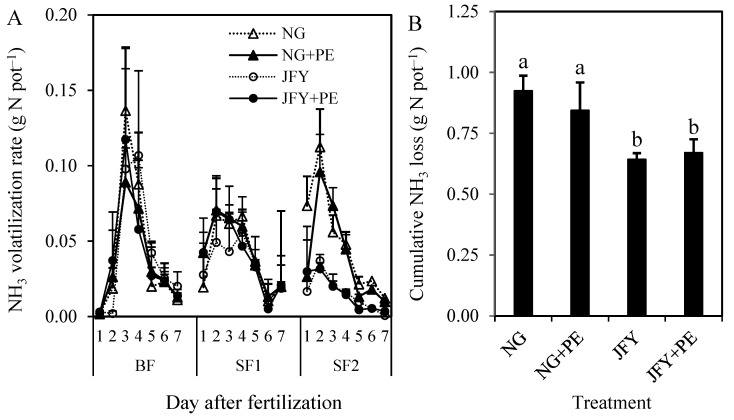
Effects of PE addition on the ammonia (NH_3_) volatilization rate (**A**) and cumulative NH_3_ loss (**B**) from paddy soil planted with a conventional rice (NG) and a hybrid rice cultivar (JFY). The BF, SF1 and SF2 refer to the basal (BF) and first and second supplementary fertilizers (SF1 and SF2), respectively. Bars represent the SD of the means (*n* = 3), and the different lowercase letters above the column indicate that the differences among treatments are significant at *p* < 0.05.

As shown in Figure 2A, the dynamics of the NH_3_ volatilization flux pattern were similar among the four experimental treatments within seven days after three N fertilizations. The NH_3_ volatilization rate increased remarkably at first and then declined gradually to a level near 0 g N pot^−1^ after N input. All treatments reached the maximum values on the second or third day. Either the rice cultivar or PE addition did not change the foregoing pattern of NH_3_ volatilization fluxes in rice paddy soil.

Data in Figure 2B show that the total NH_3_ volatilization from two hybrid rice plantations averaged significantly lower (*p* < 0.05, 25.7%) than two conventional rice plantations (0.64–0.67 vs. 0.84–0.92 g N pot^−1^). This difference was mainly noticed at the second supplementary fertilizer (SF2) observation (Table S1). The addition of PE did not affect the cumulative NH_3_ volatilization from paddy soil with conventional or hybrid rice.

**Figure 3:**
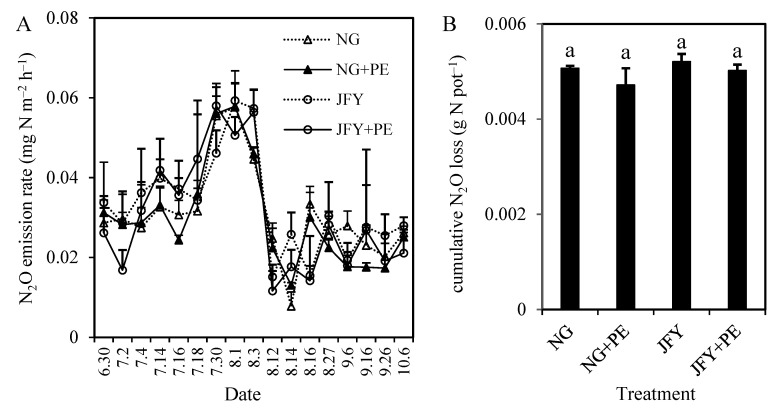
Effects of PE on the dynamics of nitrous oxide (N_2_O) emission rate (**A**) and cumulative N_2_O losses (**B**) from paddy soil planted with a conventional rice (NG) and a hybrid rice cultivar (JFY). Different bars represent the SD of the means (*n* = 3), and the same lowercase letters above the column indicate that the differences among treatments are not significant at *p* < 0.05.

For both conventional- and hybrid-rice-planted paddy soils, the N_2_O emission flux patterns were almost the same, and were not changed by PE addition or no PE addition (Figure 3A). The N_2_O production rate was low, at only 0.01–0.03 mg N m^−2^ h^−1^ during the first 20 days after rice seedling transplantation, though there were heavy inputs of N via the basal (BF) and first supplementary fertilizer (SF1). But, N_2_O emissions increased markedly when the overlying water was drained. During the mid-season drainage period, the N_2_O emission rates reached 4558 μg N m^−2^ h^−1^ for conventional rice and 46–59 μg N m^−2^ h^−1^ for hybrid rice. At the end of the mid-season drainage period, the N_2_O emission rates dropped to a low level again. Hybrid rice treatments emitted equal seasonal cumulative N_2_O to conventional rice treatments (Figure 3B). PE had no influence on the cumulative N_2_O loss from paddy soils with either conventional or hybrid rice.

### 2.4. Averaged pH, NH_4_^+^-N and NO_3_^−^-N Concentrations of Floodwater

**Figure 4:**
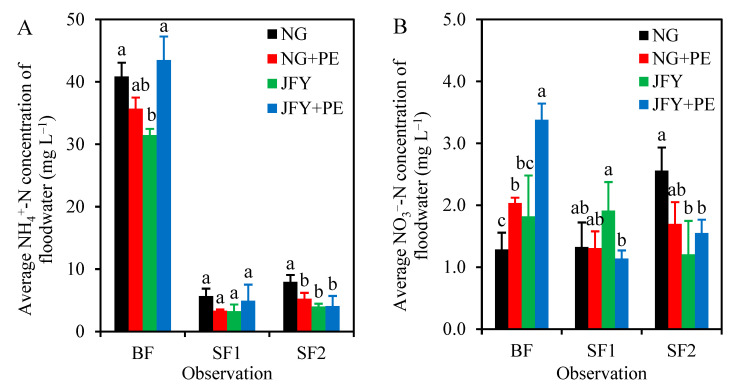
Effects of PE on the mean of floodwater NH_4_^+^-N (**A**) and NO_3_^−^-N (**B**) contents after three N fertilizations for conventional rice (NG) and hybrid rice (JFY). Bars represent the SD of the means (*n* = 3), and the different lowercase letters above the column indicate that the differences among treatments are significant at *p* < 0.05.

There was almost no difference in overlying water pH under the four treatments after each N application (Table S2). Without PE addition, the overlying water pH in the JFY treatment was, on average, lower than that in the NG treatment, especially at the BF and SF2 stage (Figure 4A). For hybrid rice, PE significantly raised the mean NH_4_^+^-N concentration of overlying water at the BF observation. During the SF1 and SF2 periods, the NH_4_^+^-N concentrations in overlying water were lower in each treatment, and the effect of microplastics on them was basically the same as that in the BF period. However, the situation changed for conventional rice compared to hybrid rice, where PE reduced the NH_4_^+^-N concentration in overlying water among all three fertilization periods and decreased significantly at the SF2 period (Figure 4A). Overall, the addition of PE significantly (*p* < 0.05) increased the average NO_3_^−^-N concentrations in the overlying water in the BF stage and had almost no effects on it during the SF2 periods for the two rice fields. However, the average NO_3_^−^-N concentrations in the JFY + PE treatment significantly (*p* < 0.05) decreased by 40.5% compared with the JFY treatment at the SF1 period. (Figure 4B).

### 2.5. Soil Properties

As shown in Table 3, PE did not significantly impact the pH, total N, NO_3_^−^-N and available phosphorus (P) contents in paddy soil planted with either conventional or hybrid rice. For soil planted with the conventional rice cultivar, NH_4_^+^-N contents decreased by 2.66 mg kg^−1^ after the PE addition. In addition, the NH_4_^+^-N and NO_3_^−^-N contents in hybrid rice were all lower than those in common rice. Microplastics significantly (*p* < 0.05) reduced the available potassium (K) by 25.0% in the paddy soil with conventional rice. With an equal N application rate, PE increased the soil organic matter (SOM) and total organic carbon (TOC) contents of soils planted with both conventional and hybrid rice, which showed that the SOM and TOC contents were increased by 7.4–9.5% and by 7.5–9.5% following the PE addition (Table 3).

### 2.6. Soil Urease Activity and nirK, nirS, nosZ Genes Abundance Copies

Microplastics exerted contrasting effects on the urease activity in soils planted with conventional and hybrid rice (Table 4). The effect was manifested as an increase in soil urease activity under hybrid rice but an inhibition under conventional rice, though there was no significant (*p* > 0.05) difference in soil urease activity whether with or without PE addition.

The copy number of *nosZ* genes in both rice paddy soils under each treatment was much higher than that of *nirK* and *nirS*, with the former being one order of magnitude higher than the latter (Table 4). Microplastics reduced the *nirK* and *nirS* gene abundance in the soils. For paddy soils grown with conventional rice, the copy number of the *nirK* and *nirS* genes was significantly (*p* < 0.05) reduced by 70.7% and 56.3% in the NG + PE treatment compared to NG treatment, respectively. For soils grown in hybrid rice, they were also significantly (*p* < 0.05) decreased by 25.8% and 75.9% in the JFY + PE treatment compared to JFY treatment, respectively. In addition, microplastics slightly increased the copy number of *nosZ* genes in soils planted with hybrid rice, but this value decreased by 45.7% for soils with common rice.

## 3. Discussion

### 3.1. Effects of Microplastic on Rice Grain Yield and Quality

Essential factors affecting rice production are soil properties, continuous improved fertilizer application methods, evolution of rice cultivars and exogenous materials addition [28]. The results in Table 1 indicate that a higher grain yield in hybrid rice than common rice may be promoted by increasing rice biomass, reservoir and material translocation capacity [29]. In this study, the plant height of hybrid rice was higher than conventional rice and ensured biomass accumulation. In addition, in [30], the reason for why hybrid rice improved rice production and reservoir capacity was a great increase in spike number, which is similar to this study. Zhang et al. (2008) found that differences in yield among various rice cultivars mostly derived from differences in N uptake and physiological utilization efficiency [31]. The N uptake and physiological utilization rates for hybrid rice were higher than those of conventional rice in our research, which exactly correspond to the yield results in Table 1.

Microplastic PE had a decreasing effect on the grain yields of both common and hybrid rice. This was comprehensively due to the negative influences of PE on the yield-related agronomic traits, including plant height, spike number and grain number per spike (Table 1). The results showed that microplastics reduced a greater yield for common rice, which may be due to the lower reduction in PEN in hybrid rice than conventional rice. Microplastics in the soil might cause mechanical damage to rice, such as changes in root length, and this breakage may also impact the long-term developmental processes of plants, thus affecting the PEN [32]. In addition, microplastics have an inhibitory effect on plant growth, leading to a lower plant height and causing yield reduction [21,33].

Crop amino acid content is an important determining factor of edible grain quality. Consequently, increasing the content of grain amino acid has been an important goal and research hotspot for rice cultivation in China [34]. Exogenous soil additives such as biochar could preserve and increase the quality and amino acid content of rice [35]. In this study, microplastics increased the total amino acid content of conventional rice grains but had no impact on hybrid rice (Figure 1). This represents that the effect of microplastics on the amino acid content is controlled by varietal characteristics [36]. A previous study found that microplastic limits the amino acid transport process [37]. Also, it restricts the common rice growth and amino acid production, which reduced the amino acid content in our research.

### 3.2. Responses of NH_3_ Volatilization to Microplastic

This study demonstrated that the dynamics trend of NH_3_ loss did not change depending on the rice cultivar and PE, and showed a tendency of first increasing and then decreasing at each fertilization observation. However, microplastics exerted different effects on the cumulative NH_3_ loss from the soils planted with conventional and hybrid rice. For common rice, microplastics reduced the cumulative NH_3_ loss, and the opposite appeared to be the case in hybrid rice. She et al. (2018) showed that NH_3_ loss was positively related to the overlying water pH and NH_4_^+^-N content [22]. In addition, Soares et al. (2012) indicated that soil urease activity (especially after fertilization) also affected NH_3_ volatilization [38].

Feng et al. (2022) found that the coexistence of biochar and microplastics led to a decrease in overlying water pH, which caused a decrease in NH_3_ volatilization [39]. In this study, there was a relationship between conventional rice NH_3_ loss and overlying water pH mainly at the BF and SF2 periods, while it was not found at the SF1 period (Table S2). The overlying water pH decreased in the BF period with microplastics, which reduced NH_3_ volatilization. Microplastics raised the overlying water pH at the BF and SF1 periods in hybrid rice but decreased it at the SF2 period. Thus, there was no significant effect on cumulative NH_3_ loss. Microplastic increased NH_4_^+^-N concentrations in the overlying water of the hybrid rice treatment but decreased them in the conventional rice treatment (Figure 4). At the BF and SF2 periods, the microplastic reduced the average NH_4_^+^-N concentrations in conventional rice floodwater by 0.5–44.5% and 20.6–65.4%, respectively, which also indicates that the NH_3_ volatilization was dominated by the direct conversion of NH_4_^+^-N to NH_3_ in the early stage of rice growth. For common rice, microplastics might have a strong sorption capacity for NH_4_^+^-N released from applied urea [39], contributing to lower NH_3_ volatilization.

Microorganisms and enzymes can promote the conversion of NH_4_^+^-N in the soil [40]. In addition, Ng et al. (2021) found that MPs in the soil altered the soil enzymes and microbiota abundance and diversity [21]. Therefore, microplastics may affect the soil microbial nitrification process and NH_3_ volatilization by impacting soil urease activity. Microplastic decreased urease activity in conventional rice grown soil but increased that in hybrid-rice-planted paddy soil (Table 4). Previously, Liu et al. (2005) found that the adsorption capacity of different rice varieties on various ions in the soil differed somewhat [41]. The large biomass of hybrid rice, coupled with microplastics, disturbed the soil structure, which could have promoted microbial colonization and stimulated the soil urease activity [40].

### 3.3. Effects of Microplastic on N_2_O Emission

Studies have shown that the main processes determining N_2_O emissions include the nitrification and denitrification of soil N [5,42]. When the paddy field was flooded, insufficient O_2_ supplication promoted N_2_O production through denitrification, and the generated N_2_O was further reduced to N_2_. During the drainage periods, the good aeration environment was conducive to simultaneous nitrification and denitrification, and the N_2_O emission increased dramatically [43]. In this study, the N_2_O emission peaks under all treatments were observed at the drainage periods, which was consistent with the research of Zou et al. (2007) [7].

Although microplastic had no significant influence on the cumulative N_2_O emissions, it can also be seen that PE suppressed the N_2_O emissions and that the suppressing effect in conventional rice was better than that in hybrid rice (Figure 3B). Our previous work in a fertile paddy field with excessive N input also found that microplastic could inhibit the N_2_O emission with common rice but the effect of it on hybrid rice was not significant [13]. It was confirmed that N_2_O could diffuse to the atmosphere through the rice plant aerenchyma system when the soil was flooded [44]. Therefore, the different effects of microplastic on N_2_O emission from paddy soils with conventional or hybrid rice might be related to the developmental status of the aeration tissues among different rice cultivars.

Microplastic reduced the gene copies of *nirK* and *nirS* in soil with both conventional and hybrid rice and mitigated the corresponding N_2_O emissions (Figure 3B). According to the previous study, the reduction in soil N_2_O emission through nitrification and denitrification processes was positively linked to *nirS* and *nirK* gene abundances, while negatively correlated with *nosZ* gene abundance [45]. This further confirms our results that microplastic reduced *nirK* and *nirS* gene copy numbers in paddy soils with both conventional and hybrid rice. The further underlying mechanisms of microplastic on N_2_O emission may be investigated in the future.

### 3.4. Microplastic Changed Soil Properties

As a solid contaminant, microplastic has remarkable impacts on soil properties [19,20]. The current results indicate that microplastics reduced the soil pH, which is consistent with the previous study [46]. Changes in soil pH have been shown to affect the effectiveness of soil nutrients, thereby affecting plant growth and crop production [47]. Soil pH affects ion morphology and quantity, and a higher pH potentially increases soil potassium fixation capacity [48]. In this study, microplastics might have inhibited the soil cation exchange process and thereby lowered the soil pH, which led to the fixation of K and ultimately reduced the available K concentration in the soil with conventional rice [46,49]. However, the effects of microplastics on the available K and available P contents in soils grown with hybrid rice were different from those with conventional rice. Microplastics increased contents of available P and K in hybrid-rice-planted paddy soils. Due to the presence of microplastics, the development of the plant root system was restricted [50], which might result in a decline in the root uptake of K and conversely increase the soil available K contents. The strong physiological characteristics of hybrid rice under the action of microplastics, together with the anaerobic environment of flooding, could cause the release of P from the decomposition of dead soil microorganisms, increasing the available P content in soils with hybrid rice [51].

Microplastics elevated the total N contents in paddy soils with both conventional and hybrid rice. This may be due to microplastics acting as an organic substrate to be used by microorganisms and consume oxygen, forming an oxygen concentration gradient on the functional bacteria inner surface that is favorable for N transformation, indirectly affecting the N cycling and increasing the soil total N content [52]. The NH_4_^+^-N concentration reduced by 45.3% in NG + PE compared to NG. Previous studies have indicated that plastic films left in the soil for a long time reduced the soil inorganic N content, which is consistent with this study [53]. Microplastics might also give the soil a higher rate of NH_4_^+^-N conversion, leading to a more rapid decrease in NH_4_^+^-N concentration [54]. In addition, microplastics reduced NH_4_^+^-N but increased NO_3_^−^-N concentration in hybrid rice. The reason for this might be that the biomass of hybrid rice was greater than conventional rice and that microplastics increased the soil porosity. Therefore, they increased the nutrient flux rate through the sediment and oxygen diffusion rate, which promoted nitrification but weakened denitrification and anaerobic NH_4_^+^-N oxidation reactions, thereby reducing NH_4_^+^-N concentration but increasing NO_3_^−^-N concentration [55,56].

Boots et al. (2019) found that microplastic decreased the SOM, resulting in a decline in soil fertility. However, we found that microplastic increased the SOM [46]. Furthermore, another study showed that microplastics from various materials had different influences on SOM content [25]. The material in this study was low-density polyethylene (LDPE), which is different from high-density polyethylene (HDPE) and biodegradable polylactic acid (PLA) that were used in Boots’ research [46]. Meanwhile, most of the microplastics were not separated from the soil, but they were still oxidized and counted [55]. The sequestration of organic carbon in agricultural soils depends mainly on the dynamic balance between the input of organic carbon and the output of its mineralization and leaching. Organic additives are often considered to promote soil carbon sequestration and therefore to enhance the soil fertility [57]. The present study showed that microplastic increases the TOC contents, and the effect was more pronounced in common rice. Long-term inorganic fertilizer application alone accelerated soil acidification and had a negative impact on the continuous and stable input of exogenous carbon and its fixation, which may be a reason [58]. In addition, the churning activity of paddy soils before transplanting under flooding conditions makes large soil particles break up to form a large amount of fine soil particles, which can use microplastics as carriers to better wrap soil particulate organic carbon through chemical adsorption and other processes, slowing down the decomposition of soil active organic carbon to a certain extent. And, the churning activity drives soil cohesion, increasing the average residence time of soil active carbon under flooding conditions, and thus increasing the TOC content [59]. Overall, microplastics could affect some key soil properties, but the mechanisms underlying these changes are still poorly understood.

### 3.5. Analysis of Interactions among Microplastic Addition and Rice Cultivar

The results in Table 5 show that rice varieties affected both grain yield and paddy soil NH_3_ volatilization. The total amino acids content was affected by rice varieties and microplastic addition. Neither rice varieties nor microplastic significantly affected N_2_O emission. The effects of microplastics on organic N degradation, nitrification and denitrification processes were also related to the type and dose of microplastics [60,61]. This study focuses on a single microplastic type and dose. Hence, more types and addition rates should be considered when studying how microplastics affect N_2_O emissions in further research.

## 4. Materials and Methods

### 4.1. Background Information

We collected the tested soils from a typical >10 year reclaimed costal saline field with rice–wheat rotation located at Dafeng (33°20′ N, 120°47′ E), Jiangsu Province, China. This area has a subtropical monsoon climate, where the average annual air temperature and rainfall were 14.4 °C and 1067 mm, respectively. The top layer (0–20 cm) of soil samples was collected from five sites in an approximately 0.5 ha paddy field. The soil sample was mixed and air-dried for approximately two weeks. Thereafter, we ground the soils and sieved them through a 2 mm nylon sieve, and then refilled them in layers into soil pots (inner diameter 30 cm, height 28 cm) with approximately the same bulk density as in the field. Each soil column contained about 20 kg of soil. The tested soil was classified as salinized fluvo-auic soil and some selected properties of 0–20 cm topsoil were as follows: pH 8.05 (soil–water ratio 1:5), soil organic matter (SOM) 7.7 g kg^−1^, total N 0.41 g kg^−1^, NH_4_^+^-N 1.13 mg kg^−1^, NO_3_^−^-N 0.35 mg kg^−1^, Olsen-P 9.13 mg kg^−1^ and available K 64.5 mg kg^−1^. Low-density polyethylene (LDPE) was applied in the experiment as the tested microplastic, which was sourced from Yangli Chemical Company, Shanghai, China. The main properties of it were as follows: density 0.92 g cm^−3^, particle size < 23 µm and melting point 105 °C.

### 4.2. Experimental Treatments and Rice Management

Two rice cultivars were planted in the current study, which were conventional (Nangeng 5055, NG) and hybrid (Jiafengyou 6, JFY), respectively. For each rice cultivar, two treatments (one with PE and another without PE) with three replications were evaluated. Therefore, we abbreviated the four experimental treatments as NG, NG + PE, JFY and JFY + PE, respectively. In addition, no N and PE application was labeled as the control in order to calculate the N utilization efficiencies of rice. We mixed 60 g PE with the 20 kg soil when being repacked into each soil column (pot) [62]. The total fertilizer N application (270 kg N ha^−1^, equal to 4.15 g fertilizer N pot^−1^) was split into a basal fertilizer (BF) at transplanting (30%, BF) and the first (30%, SF1) and second (40%, SF2) supplementary fertilizers. Phosphorus (P) and potassium (K) fertilizers were applied in forms of calcium superphosphate (12% P_2_O_5_) and potassium chloride (60% K_2_O) at rates of 100 kg P_2_O_5_ ha^−1^ and 300 kg K_2_O ha^−1^. Both P and K fertilizers were applied as BF in all treatments Rice was transplanted on 28 June 2021 (three hills per pot, with two conventional rice seedlings per hill but with one hybrid rice seedling per hill) and then harvested on 22 October 2021. The drainage periods were on 28 July to 9 August and 17 October to 24 October 2021.

### 4.3. Sampling and Measurements

#### 4.3.1. Crop

After rice harvest, we measured and recorded the plant height, spike number and kernels per spike of rice sampled from each pot. Meanwhile, we calculated thousand grain weight. The dried weight of straw and grain was measured. The sample of rice straw and grain was crushed and passed through a 0.2 mm sieve to measure total N content. Rice straw or grain was digested in a H_2_SO_4_-H_2_O_2_ mixture and the total N content was determined by the Kjeldahl method [63]. The N use efficiencies were estimated by the following formulas [64]:

(1) $$\mathrm{Agronomic}\;\mathrm{efficiency}\;\mathrm{of}\;N\;(\mathrm{AEN},\;g\;\mathrm{grain}/g)=\frac{C_{F}-C_{0}}{N}$$

(2) $$\mathrm{Recovery}\;\mathrm{efficiency}\;\mathrm{of}\;N\;\left(\mathrm{REN},\;\%\right)=\frac{N_{F}-N_{0}}{N}\times 100$$

(3) $$\mathrm{Physiological}\;\mathrm{efficiency}\;\mathrm{of}\;N\;(\mathrm{PEN},\;g\;\mathrm{grain}/g)=\frac{\mathrm{AEN}}{\mathrm{REN}}$$

(4) Partial factor productivity (PFPN, g grain/g)

$C_{F}$ and $N_{F}$ refer to the rice yield (g pot^−1^) and N uptake capacity (%) of rice planted in fertilizer N (urea)-applied treatments after harvest; $C_{0}$ and $N_{0}$ refer to the rice yield (g pot^−1^) and N uptake capacity (%) of rice planted in the CK treatment after harvest; N refers to the inorganic N fertilizer (urea)-applied rate.

The samples for determining the amino acid content of rice were the grain crushed and passed through a 60-mesh sieve. An amino acid analyzer (Hitachi L-8800, Tokyo, Japan) was used to determine the amino acid content of the grain samples.

#### 4.3.2. NH_3_ Volatilization

Daily NH_3_ volatilization rate was monitored using the sponge absorption method [65]. The sampling device was a polyvinylchloride cylindrical plastic tube (15 cm in height and 15 cm in inner diameter). Two sponges (2 cm in thickness and 16 cm in diameter) were placed at the top and 5 cm from the bottom of the tube, and were soaked in 15 mL of phosphoglycerin. The lower sponge was taken out as the sample and replaced with a new sponge. The sampled sponges were extracted with 300 mL of 1 mol/L KCl solution and shaken for 1 h. The NH_4_^+^-N content was measured by the indophenol blue colorimetry method. The NH_3_ volatilization was estimated according to the following formula:

(5) $$\omega =\frac{m\times V_{m}\times V_{e}}{V_{s}}\times 10^{-3}$$

ω: Ammonia content in a single ammonia volatilization collection device (mg);

m: NH_4_^+^-N concentration (mg L^−1^);

$V_{m}$: The volume of solution used to measure absorbance after constant volume (mL);

$V_{e}$: KCl solution volume for extracting ammonia from sponge (mL);

$V_{s}$: The volume of extracting solution used for measurement (mL).

#### 4.3.3. N_2_O Emission

The gas sampling for the determination of N_2_O was carried out using the modified closed chamber method as described in [66]. The chamber was a Plexiglas cylinder (100 cm in height and 36 cm in inner diameter) covered with Al foil. The gas in the chamber was mixed by a fan at the top. Water was fitted into the grove at the bottom to form a sealed space when collecting gas.

For each N fertilizer period and drainage period, gas sampling was conducted on the second, fourth, sixth and eighth days. During other rice growth periods, it was collected every ten days. The gas sampling was generally arranged between 6:00 and 8:00 a.m. and 18.5 mL gas sample was collected at 0, 15, 30 and 45 min after being sealed. The N_2_O concentrations were measured with a gas chromatograph (Agilent 7890B, Agilent Technologies, Santa Clara, CA, USA), and then the cumulative N_2_O emission was calculated.

#### 4.3.4. Floodwater and Soil pH, NH_4_^+^-N and NO_3_^−^-N

Floodwater was collected on the first, third, fifth and seventh days just after N fertilizations (BF, SF1 and SF2). After rice harvest, 0–20 cm topsoil samples were collected and were frozen at −20 °C for NH_4_^+^-N and NO_3_^−^N analysis, and −80 °C for molecular analysis. The floodwater pH was measured by in situ measurement during three N fertilizations and soil pH was determined in the 1: 2.5 (*w*/*v*) soil/water ratio. Approximately 10 g soil samples were extracted with 50 mL KCl solution (2 mol/L), and then indophenol blue colorimetry and an ultraviolet spectrophotometer were used for measuring NH_4_^+^-N and NO_3_^−^-N concentrations.

#### 4.3.5. Other soil Properties

The contents of soil organic carbon (SOC) and organic matter (SOM) were measured by potassium dichromate methods. We used the bicarbonate extraction–molybdenum antimony colorimetric method to determine available P, NH_4_^+^-N acetate extraction–flame photometry to determine available K and Kjeldahl method to determine total N [63].

#### 4.3.6. Soil Urease Activity and Microbial Abundance

Soil urease activity was determined by indophenol blue colorimetry. Fresh soil (air-dried and passed through 1 mm sieve) was incubated with a mixture of toluene, urea solution and citric acid buffer at 37 °C for 24 h. Subsequently, we added the sodium phenol and sodium chlorate, and determined the absorbance at 630 nm by enzyme standard instrument (EPOCH 2). Finally, the soil urease activity was calculated according to the absorbance.

The *nirK*, *nirS* and *nosZ* gene abundances of topsoil (0–20 cm) samples at harvest were measured by Shanghai Majorbio Biomedical Co., Ltd. according to the processes detailed in publication of Ye et al. (2021) [5].

### 4.4. Data Statistics

We performed the statistical analysis with the SPSS 16.0 software. One-way ANOVA was used to test the influence of different treatments on response factors. Two-way ANOVA was applied to analyze the interaction between two variables (rice cultivar and microplastic). Means of all treatments were compared by Duncan’s multiple range test and the differences were considered as significant at the 95% level (*p* < 0.05).

## 5. Conclusions

Microplastic can affect rice growth, grain yield and quality, N utilization efficiency, reactive N losses (NH_3_ and N_2_O) and soil properties. It decreased the REN, AEN, PEN and PFPN, leading to a significant reduction for common and hybrid rice grain production. However, PE had no significant influence on grain yield. Furthermore, PE had generally negative effects on rice growth and the yield components, which comprehensively contributed to the relative lower grain yield of rice when exposed to PE. Though there was almost no influence on the total amino acid content for hybrid rice grain, PE significantly increased the total amino acid content in conventional rice grain. In addition, PE enhanced the SOM and TOC content in the soil planted with conventional and hybrid rice. Microplastic raised NH_3_ volatilization from soils with conventional rice but had no effects on NH_3_ volatilization from soils with hybrid rice. Lowering the pH in the overlying water and enhancing the adsorption of NH_4_^+^-N, as well as its nitrification, could be the underlying mechanism for reducing NH_3_ volatilization after the addition of microplastic. PE had some inhibitory effects on N_2_O emissions. A decrease in the *nirK* and *nirS* gene copy number resulted in the mitigating effect of microplastic on N_2_O emissions.

## Figures and Tables

**Table 1:** Effects of PE input on the plant height, straw biomass and rice yield, as well as its components of conventional rice (NG) and hybrid rice (JFY).

Treatment	Plant Height	Straw Biomass	Grain Yield	Spike Number	Kernels per Spike	Thousand Grain Weight (g)
	(cm)	(g/pot)	(g/pot)			
NG	71.5± 3.5 ab	29.2 ± 3.2 b	52.5 ± 0.5 b	22 ± 1 a	105 ± 11 b	24.5 ± 1.0 a
NG + PE	67.2 ± 2.5 b	32.5 ± 3.1 ab	48.2 ± 4.8 b	21 ± 2 a	110 ± 10 b	24.8 ± 0.1 a
JFY	78.2 ± 4.1 a	34.9 ± 2.6 ab	66.2 ± 4.7 a	16 ± 1 b	225 ± 12 a	21.0 ± 0.7 b
JFY + PE	74.3 ± 3.9 a	36.8 ± 3.2 a	61.6 ± 5.0 a	18 ± 1 b	220 ± 13 a	22.1 ± 0.3 b

Note: Data are presented as mean ± SD (*n* = 3). Different lowercase letters within each column indicate that the differences among treatments are significant at *p* < 0.05.

**Table 2:** Effects of PE on nitrogen (N) use efficiencies of conventional rice (NG) and hybrid rice (JFY).

Treatment	N Recovery Efficiency	N Agronomic Efficiency	N Physiology Efficiency	Partial Factor Productivity of N
	(REN)	(AEN)	(PEN)	(PFPN)
	%	g grain g^−1^ N		
NG	17.8 ± 1 a	10.1 ± 0.1 a	57.0 ± 2.1 b	12.7 ± 0.1 b
NG + PE	17.4 ± 2 a	9.10 ± 1.2 a	52.3 ± 0.7 c	11.6 ± 1.2 b
JFY	18.5 ± 2 a	12.2 ± 1.9 a	65.9 ± 2.4 a	16.0 ± 1.9 a
JFY + PE	17.3 ± 4 a	11.1 ± 2.8 a	64.0 ± 2.3 a	14.9 ± 2.8 ab

Note: Data are presented as mean ± SD (*n* = 3). Different lowercase letters within each column indicate that the differences among treatments are significant at *p* < 0.05.

**Table 3:** Effects of PE on selected soil properties under the paddy soil planted with common rice (NG) and hybrid rice (JFY).

Treatment	Soil pH	Total N	Ammoniu-m N	Nitrate N	Available P	Available K	Soil Organic Matter	Total Organic Carbon
		(g kg^−1^)	(NH_4_^+^-N, mg kg^−1^)	(NO_3_^−^-N, mg kg^−1^)	(mg kg^−1^)	(mg kg^−1^)	(SOM, g kg^−1^)	(TOC, g kg^−1^)
NG	7.94 ± 0.06 a	0.50 ± 0.01 a	5.87 ± 0.80 a	0.92 ± 0.18 a	9.55 ± 0.4 a	115.5 ± 5.50 a	7.46 ± 0.36 b	4.33 ± 0.21 b
NG + PE	7.91 ± 0.04 a	0.51 ± 0.01 a	3.21 ± 0.50 b	0.78 ± 0.11 a	9.29 ± 0.9 a	86.67 ± 7.77 b	8.17 ± 0.40 a	4.74 ± 0.23 a
JFY	7.96 ± 0.02 a	0.48 ± 0.01 a	2.70 ± 0.66 b	0.37 ± 0.19 b	8.56 ± 1.1 a	84.33 ± 5.13 b	7.33 ± 0.24 b	4.25 ± 0.14 b
JFY + PE	7.94 ± 0.04 a	0.49 ± 0.03 a	2.65 ± 0.13 b	0.40 ± 0.26 b	8.75 ± 1.2 a	87.33 ± 4.04 b	7.87 ± 0.27 ab	4.57 ± 0.16 ab

Note: Data are presented as mean ± SD (*n* = 3). Different lowercase letters within each column indicate that the differences among treatments are significant at *p* < 0.05.

**Table 4:** Changes in urease activity and the abundances of *nirK*, *nirS* and *nosZ* genes in soils sampled at harvest as influenced by PE and rice cultivars (NG and JFY).

Treatment	Urease Activity	*nirK*	*nirS*	*nosZ*
	U g^−1^ soil	×10^5^ copies g^−1^ soil		
NG	86.5 ± 8.7 ab	9.9 ± 1.7 a	6.4 ± 0.1 a	51.0 ± 31.5 a
NG + PE	72.2 ± 8.5 b	2.9 ± 1.0 b	2.8 ± 1.1 b	27.7 ± 21.6 a
JFY	89.1 ± 8.5 ab	3.1 ± 1.0 b	2.9 ± 0.4 b	38.6 ± 13.4 a
JFY + PE	90.4 ± 9.0 a	2.3 ± 1.3 b	0.7 ± 0.4 c	40.4 ± 24.9 a

Note: Data are presented as mean ± SD (*n* = 3). Different lowercase letters within each column indicate that the differences among treatments are significant at *p* < 0.05.

**Table 5:** Two-way ANOVA for the effects of microplastic (PE) and rice cultivar on grain yield, total NH_3_ volatilization, total N_2_O emission and total amino acid contents in rice grain (*p* value).

Factor	Grain Yield	Total NH_3_ Volatilization	Total N_2_O Emission	Total Amino Acid Contents
PE	0.235 ^ns^	0.539 ^ns^	0.049 ^ns^	0.048 *
Cultivar	0.005 **	0.001 **	0.098 ^ns^	0.001 **
PE × Cultivar	0.971 ^ns^	0.232 ^ns^	0.511 ^ns^	0.083 ^ns^

Notes: * *p* < 0.05; ** *p* < 0.01; ns refers to not significant.

## Data Availability

All data are included in the main text.

## Supplementary Materials

The following supporting information can be downloaded at: https://www.mdpi.com/article/10.3390/plants13091279/s1. Table S1: Effects of microplastic polyethylene (PE) on the ammonia (NH_3_) volatilization from paddy soil planted with common rice cultivar Nangeng 55 (NG) and hybrid rice cultivar Jiafengyou 6 (JFY); Table S2: Effects of microplastic polyethylene (PE) addition on mean pH of floodwater observed after each application of inorganic nitrogen fertilizer urea.
