# Supplementary material for: Microplastic Has No Effect on Rice Yield and Gaseous N Emission from an Infertile Soil with High Inorganic N Inputs
Source: Plants (Basel). 2024 May 6;13(9):1279. doi: 10.3390/plants13091279 (PMC11085246; doi:10.3390/plants13091279)
Supplement: Supplementary file 1 [file plants-13-01279-s001.zip › plants-2936726-supplementary.pdf]

Supplementary Materials:

Table S1: Effects of microplastic polyethylene (PE) on the ammonia (NH_3) volatilization from paddy soil planted with common rice cultivar Nangeng 55 (NG) and hybrid rice cultivar Jiafengyou 6 (JFY).

Treatment	Cumulative NH_3 volatilizations (g N pot^{-1})		
	Basal fertilization	The first supplementary fertilization	The second supplementary fertilization
NG	0.30±0.08 a	0.28±0.05 a	0.35±0.15 a
NG+PE	0.25±0.13 a	0.31±0.07 a	0.28±0.12 a
JFY	0.30±0.07 a	0.24±0.10 a	0.11±0.03 b
JFY+PE	0.28±0.14 a	0.28±0.07 a	0.11±0.03 b

Note: Data are presented as mean±SD (n = 3). Different lowercase letters within each column indicate the differences among treatments are significant at $p < 0.05$.

Table S2: Effects of microplastic polyethylene (PE) addition on mean pH of floodwater observed after each application of inorganic nitrogen fertilizer urea

Treatment	Basal fertilization	First supplementary fertilization	Second supplementary fertilization
NG	8.09 ± 0.21 a	8.33 ± 0.21 a	8.49 ± 0.21 a
NG+PE	7.96 ± 0.23 a	8.48 ± 0.28 a	8.47 ± 0.25 a
JFY	8.16 ± 0.17 a	8.40 ± 0.15 a	8.37 ± 0.25 a
JFY+PE	8.23 ± 0.16 a	8.48 ± 0.31 a	8.21 ± 0.15 a

Note: NG, the common rice cultivar Nangeng 5055; JFY, the hybrid rice cultivar Jiafengyou 6. Data are presented as mean ± SD (n = 3). Data within each column followed by the same lowercase letter are not significantly different at $p < 0.05$.
